# Generating Virtual Short Tau Inversion Recovery (STIR) Images from T1- and T2-Weighted Images Using a Conditional Generative Adversarial Network in Spine Imaging

**DOI:** 10.3390/diagnostics11091542

**Published:** 2021-08-25

**Authors:** Johannes Haubold, Aydin Demircioglu, Jens Matthias Theysohn, Axel Wetter, Alexander Radbruch, Nils Dörner, Thomas Wilfried Schlosser, Cornelius Deuschl, Yan Li, Kai Nassenstein, Benedikt Michael Schaarschmidt, Michael Forsting, Lale Umutlu, Felix Nensa

**Affiliations:** 1 Institute of Diagnostic and Interventional Radiology and Neuroradiology, University Hospital Essen, 45147 Essen, Germany; aydin.demircioglu@uk-essen.de (A.D.); jens.theysohn@uk-essen.de (J.M.T.); axel.wetter@uk-essen.de (A.W.); nils.doerner@uk-essen.de (N.D.); Thomas-Wilfried.Schlosser@uk-essen.de (T.W.S.); cornelius.deuschl@uk-essen.de (C.D.); yan.li@uk-essen.de (Y.L.); kai.nassenstein@uk-essen.de (K.N.); benedikt.schaarschmidt@uk-essen.de (B.M.S.); michael.forsting@uk-essen.de (M.F.); lale.umutlu@uk-essen.de (L.U.); felix.nensa@uk-essen.de (F.N.); 2 Department of Neuroradiology, University Hospital Bonn, 53127 Bonn, Germany; alexander.radbruch@ukbonn.de; 3 Institute for Artificial Intelligence in Medicine, University Hospital Essen, 45147 Essen, Germany

**Keywords:** spine, magnetic resonance imaging, computing, medical informatics, machine learning

## Abstract

Short tau inversion recovery (STIR) sequences are frequently used in magnetic resonance imaging (MRI) of the spine. However, STIR sequences require a significant amount of scanning time. The purpose of the present study was to generate virtual STIR (vSTIR) images from non-contrast, non-fat-suppressed T1- and T2-weighted images using a conditional generative adversarial network (cGAN). The training dataset comprised 612 studies from 514 patients, and the validation dataset comprised 141 studies from 133 patients. For validation, 100 original STIR and respective vSTIR series were presented to six senior radiologists (blinded for the STIR type) in independent A/B-testing sessions. Additionally, for 141 real or vSTIR sequences, the testers were required to produce a structured report of 15 different findings. In the A/B-test, most testers could not reliably identify the real STIR (mean error of tester 1–6: 41%; 44%; 58%; 48%; 39%; 45%). In the evaluation of the structured reports, vSTIR was equivalent to real STIR in 13 of 15 categories. In the category of the number of STIR hyperintense vertebral bodies (*p* = 0.08) and in the diagnosis of bone metastases (*p* = 0.055), the vSTIR was only slightly insignificantly equivalent. By virtually generating STIR images of diagnostic quality from T1- and T2-weighted images using a cGAN, one can shorten examination times and increase throughput.

## 1. Introduction

The spine is one of the body regions that is the most frequently examined in MRI. Reasons for MRI are mainly back pain, sensitivity impairments, and paralysis [1,2]. To visualize the most common pathologies, short tau inversion recovery images (STIR) are often used, along with T1- and T2-weighted images. The STIR contrasts are particularly useful in the diagnosis of acute pathologies, such as inflammation or acute vertebral fractures. In the example of a vertebral body fracture, STIR is used to detect a vertebral edema and thus often enables a therapy-relevant differentiation between new and old fractures. Apart from that, the STIR sequence can lead to the decision of whether a contrast agent administration is required [3]. This is especially important considering the continuously increasing number of MRI examinations worldwide [4]. However, the acquisition of STIR sequences requires a significant amount of scanning time of three minutes [5] and is therefore susceptible to motion artifacts. In recent years, the introduction of new techniques based on deep learning has enabled advances in image processing that were previously widely considered impossible. For image processing, the use of generative adversarial networks (GAN) has become the predominant approach. As a result, it could be demonstrated that GANs are highly effective in CT denoising [6] and in inserting virtual contrast media in non-contrast MRI [7].

The aim of the present study was to generate virtual STIR (vSTIR) sequences from non-contrast non-fat-suppressed sagittal T1- and T2-weighted sequences using a cGAN and to validate these synthetic images in blinded A/B-tests on clinical MR examinations of the spine against experienced radiologists.

## 2. Material and Methods

### 2.1. Network Architecture and Preprocessing

Each scan was preprocessed by converting it into a 16-bit PNG image. The size of each slice was, in general, 512 × 512 px; in the few cases where the slice was larger, a central crop was performed across the entire scan. If the slices were smaller, the images were either padded with black to the required size of 512 × 512 px or, if the height was smaller than 256 px, dropped from the training set.

The T1, T2 images were used as input images. Additionally, a contrast limited adaptive histogram equalized filter [8] (size 32 × 32, clip limit 1.0) was applied to the T2 image and added as another channel of the input image. The intensities of all input images were rescaled to −1..1.

The Pix2PixHD framework was employed, as it has exhibited excellent performance in image-to-image tasks [9]. It is a conditional generative adversarial network using a combination of two residual networks, which are called local and global generators. The global generator produces lower-resolution images that are enhanced by a local generator. The architecture Pix2PixHD network was not changed for this study. As the output images were single-channel 16-bit vSTIR, the last layer of the network was modified to produce such output. The feature matching (VGG) part of the loss function was adapted by simple averaging to work with gray-scale images, as this loss is defined on RGB images only. The network was trained for 300 epochs, and all other parameters were left at their default (learning rate 0.0002, Adam optimizer with momentum 0.5).

### 2.2. MRI

The MRIs were performed on 1.5T and 3T MRI machines (MAGNETOM Symphony, MAGNETOM Sonata, MAGNETOM Avanto, MAGNETOM Aera, MAGNETOM Skyra) from a single vendor (Siemens Healthineers AG, Erlangen, Germany) between 2007 and 2019 at a single center (Table 1). All MRI examinations contained a sagittal non-contrast, non-fat-suppressed T1 and T2 as well as a STIR sequence with a matching field of view. The MRI scan parameters are illustrated in the supplementary material Supplementary Tables S1–S3.

### 2.3. Dataset

Using our clinical PACS, a set of 980 MRI examinations of the spine from the years 2007–2019 were identified for this study. All scans were curated by removing scans with incomplete series, non-matching T1, T2, and STIR sequences (e.g., STIR was not sagittal). The scans were then visually inspected by an experienced radiologist to ensure that no misalignment between the scans was present, resulting in 753 scans with T1/T2 and STIR images of 637 patients that were finally selected for training. For validation, two datasets were assembled, whose minimum size was previously calculated with a power analysis. For the power analysis, a two-sided equivalence test was performed with a statistical significance alpha of 0.05. A power calculation [10] with a power of 0.8, an accepted equivalence limit of the difference between the two procedures with a delta of 0.1 and expected confusions of p01 and p10 of 0.05, resulting in a minimum sample size of *n* = 86. For the first cohort, which should be evaluated in an A/B-test to verify whether the vSTIR is identifiable by a radiologist, 100 studies were randomly selected that were not part of the training cohort.

However, with this sample size, it is possible that certain pathologies are not sufficiently represented. Therefore, the second validation cohort was designed so that at least 20 studies with the most important pathologies (bone metastases, myelopathy, acute vertebral fractures, spondylodiscitis, epidural abscess, intraspinal masses, and muscular lesions) were represented. Furthermore, at least 20 healthy patients were included to check whether pathologies were inserted by our GAN [11]. In total, the cohort, which met all of the above-mentioned requirements, comprised 141 studies (Table 2). None of these studies were part of the training cohort. The distribution of the pathologies among the validation cohort is illustrated in Table 3.

### 2.4. Validation

**Figure 1:**
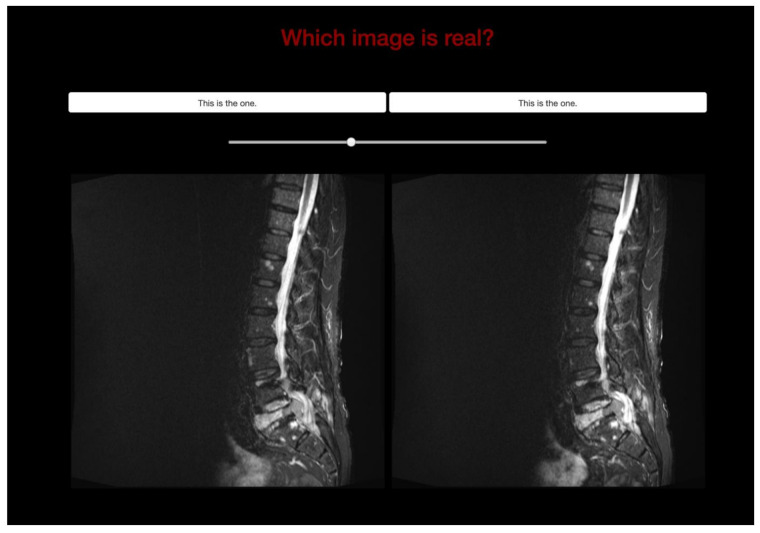
A/B-test to identify the real STIR sequence.

Two different validations were used. First, to determine whether the vSTIR was visually distinguishable from the real STIR (rSTIR), 100 MRI series from 100 distinct patients were presented to six senior radiologists in independent A/B-testing sessions. In each case, both STIR and vSTIR series were randomly demonstrated (Figure 1), and the radiologist was asked to identify the rSTIR sequence.

**Figure 2:**
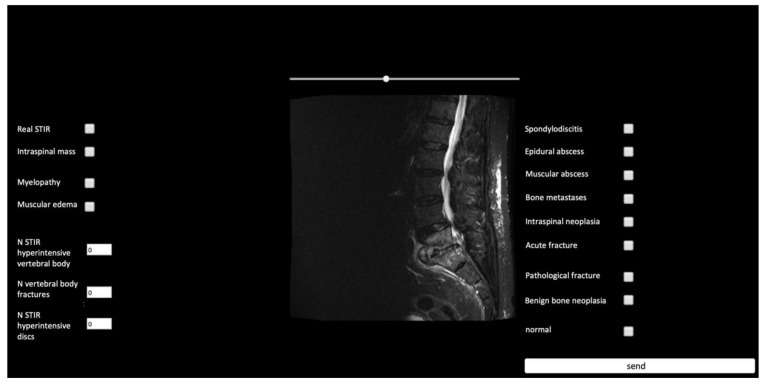
Structured reporting using our web tool developed in-house.

Second, to validate whether pathologies were represented qualitatively and quantitatively correct by vSTIR images, the validation dataset comprising 141 STIR sequences from 131 distinct patients was presented. Without knowing which series was presented to them, the radiologists were asked to perform a structured assessment of the pathological findings (Figure 2). The readers did not get any information about the STIR sequence (virtual/real) and no clinical information about the patient. Eventually, each vSTIR and rSTIR sequence was assessed by three different senior radiologists. This number of readers was chosen in order to calculate the mean and standard deviation for all quantitative findings for both the virtual STIR and the real STIR. The structured reports were compared independently for each pathology to determine whether the vSTIR was diagnostically equivalent to rSTIR. For this purpose, the number of collapsed vertebral bodies, the number of vertebral bodies with edema, and the number of STIR hyperintense discs were reported as ordinal values. Additionally, the testers were asked to determine whether it was a rSTIR and whether the following pathologies and findings were present: intraspinal mass, myelopathy, muscular edema, muscular abscess, epidural abscess, spondylodiscitis, bone metastases, intraspinal neoplasia, acute traumatic fracture, pathological fracture, or benign bone neoplasia. Finally, the testers were asked to determine whether the case was normal.

Both validation steps were performed using a generic framework for A/B-testing developed in-house (Figure 1 and Figure 2). The validation images were processed the same way as the training data. For display purposes, all images were rescaled to the intensity values 0–65,535.

### 2.5. Statistical Analysis

The ordinal values were converted to three categories: none, low (1–2), or high (>2).

Ground truth for the rSTIR ratings was determined as the median of the three ratings. Similarly, the vSTIR ratings were gathered. An equivalence test for categorical data was used to compare the ratings [12], while an equivalence test of proportions was used for binary outcomes, where the procedure of Liu 2002 [10] was employed. Inter-rater agreements, following Cohn, were computed.

A one-sided Fisher test was employed to determine whether each rater was able to distinguish the real and virtual images. Fleiss’ kappa was used to determine the inter-rater agreement.

All statistical tests were computed using R 3.6 and the irr library.

## 3. Results

**Figure 3:**
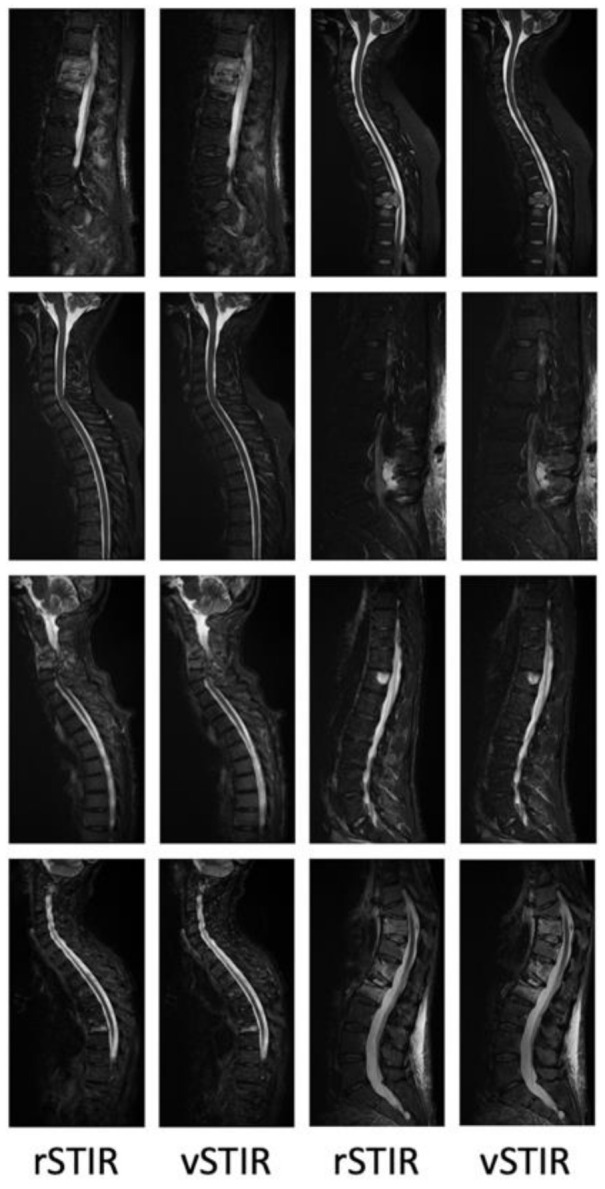
Examples of the validation cohort.

For the A/B-test only, two of the six raters showed a statistically significant tendency to be able to distinguish virtual from real images. However, the error rate was rather high in both cases (39% and 41%) and the inter-rater agreement was quite low = −0.03 (*p* = 0.25). In 34% of the cases, the raters disagreed (i.e., three raters chose the rSTIR while the other three raters chose the vSTIR), while in 41% of the cases, a majority (i.e., five or six raters) chose the rSTIR, but in 25% of the cases, they chose the vSTIR. Overall, the testers were only marginally better than a coin toss, and a single tester was even worse than an average coin toss. The results of the individual testers are indicated in Table 4. Several examples of the validation cohort are shown in Figure 3.

The analysis of the structured reports revealed that the vSTIR is equivalent to the rSTIR in 13 of 15 categories (Table 5). The two categories where the vSTIR was not equivalent to the rSTIR were the number of STIR hypertense vertebral bodies and the diagnosis of bone metastases. With a *p*-value of 0.08 for the number of STIR hyperintense vertebral bodies and 0.055 for the diagnosis of bone metastases, both categories were only slightly not significantly equivalent. In the category of detecting the true STIR, an average detection rate of only 57% was found with a very low inter-rater agreement of 0.01–0.02, consistent with the previous A/B-test.

Mean STIR/vSTIR represents how often the pathology was identified, on average, in the images. The inter-rater agreements describe how often the raters were in agreement for a given pathology, and the *p*-value measures whether the value was significantly different from 0 (i.e., no agreement at all). The significance for the difference tests whether both inter-rater agreements were significantly different. For the equivalence tests, the null hypothesis is that there is a difference between STIR and vSTIR, while the alternative hypothesis is that of their equivalence.

**Figure 4:**
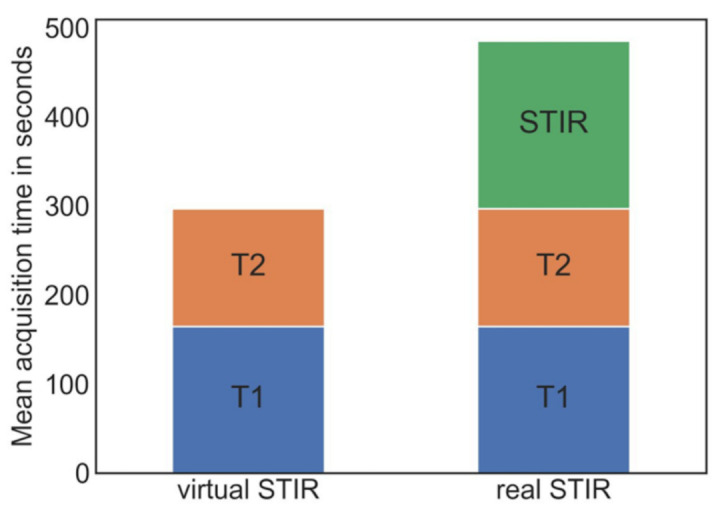
Diagram of the mean acquisition times with (left) and without STIR sequence.

**Figure 5:**
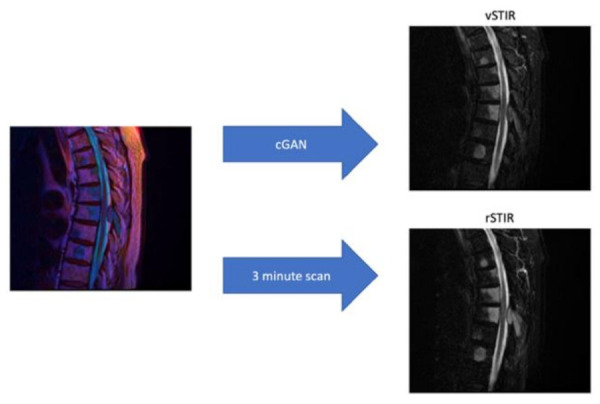
Acquisition of the virtual STIR image from the merged T1 and T2 image compared to the real STIR sequence.

To calculate the time saved by generating the vSTIR sequence, the acquisition time of the T1, T2, and STIR sequences was extracted from the DICOM header. The acquisition of the STIR sequence took 188.5 ± 46.7 s, on average, in comparison to 164.6 ± 48.1 s of T1 scan time and 132.2 ± 40.3 s of T2 scan time (Figure 4 and Figure 5).

## 4. Discussion

The aim of the present study was to generate virtual STIR (vSTIR) sequences from non-contrast non-fat-suppressed sagittal T1- and T2-weighted sequences using a cGAN and to validate these synthetic images in blinded A/B-tests on clinical MR examinations of the spine against experienced radiologists. With this approach, we were able to generate high quality synthetic STIR images that could not be distinguished from the real images even by experienced radiologists in a blinded A/B-test. In addition, a qualitative and quantitative evaluation of the pathologies depicted on the sequences showed no relevant difference between synthetic and real images, although there was a relatively high inter-rater variability.

Applications based on artificial intelligence have demonstrated a high potential in a variety of medical applications, such as the prediction of tumor histology [13], the detection of lung nodules [14], or the artifact reduction in PET imaging [15]. At the same time, there are few applications that increase time efficiency in the daily business of radiological image acquisition even though this is in great demand, considering the continuously increasing numbers of MRI examinations worldwide [16].

In this study, we developed a method to generate STIR images from non-fat-suppressed T1 and T2 images using a cGAN to reduce the scan time and the recall rate for a spinal MRI. For this purpose, a paired image-to-image translation was used [9], as this offers, on the one hand, a higher accuracy in comparison to an unpaired approach [17], and, on the other hand, the more efficient monitoring of the training cohort.

This is especially important when considering the possible dangers of a completely unsupervised cohort. In this context, Cohen et al. were able to demonstrate that pathologies can be artificially inserted or removed by using an unpaired image-to-image conversion by training the network with an imbalanced cohort [11]. However, the use of a system based on image pairs also offers uncertain risks, as it is known that GANs can produce their own artifacts, such as checkerboard artifacts [18].

Therefore, we qualitatively and quantitatively evaluated the similarity of the vSTIR images to rSTIR images. When vSTIR and rSTIR images were directly compared in an A/B-test, six consultant radiologists—each with at least seven years’ experience in musculoskeletal imaging—were not able to predict the rSTIR images.

At the same time, several studies have already indicated that image data generated by GANs can look deceptively real without representing reality [19,20]. After demonstrating that the generated STIR sequence looks real, it was therefore important to reveal that the sequence also reflects reality.

For this reason, we have had the examinations assessed by experienced radiologists in blinded A/B-tests with regard to the pathologies depicted. In this analysis, the vSTIR was equivalent to the rSTIR in 13 of 15 categories. Very similar values were indicated in two other categories—number of STIR hyperintense vertebral bodies and diagnosis of bone metastases—with the values rated statistically only slightly not significantly equivalent. On the one hand, the lack of significance could be a product of chance due to the variance between the evaluators. Alternatively, in a few cases, the vSTIR may not equivalently correspond to the rSTIR in these two categories. In the end, this should be tested in a prospective trial with a larger number of patients.

To evaluate how much time is saved by the vSTIR, the average acquisition times of T1, T2, and STIR images were compared. For an average spine MRI—which consists of a sagittal T1, T2, and STIR—about three of eight minutes of scan time could be saved. This time saving can increase the number of patients that can be scanned with one device by about one third, which significantly improves the cost efficiency of the system. In the future, this method could be combined with GAN-based compressed sensing [21] to further speed up the MRI to cope with the increasing MRI demand.

A similar model for converting a T1 or T2 sequence into a STIR sequence has been developed by Galbusera et al. [22]. In comparison, they archived very mixed results for different pathologies. This may be based on the fact that both T1 and T2 images contain independent information [23]; combining those naturally leads to an increase in information for different pathologies. To date, the only publication that combines T1 and T2 to generate a STIR sequence using deep learning was recently published by Kim et al. [24]. With only 12 healthy volunteers, this study demonstrated that deep learning can be used to generate real-looking STIR images of a knee MRI. However, it could not be demonstrated whether this virtual STIR sequence also depicts clinical reality and correctly represents pathologies. Therefore, our study is the first to generate a virtual STIR sequence with a large cohort of 657 patients, which is significantly equivalent to a rSTIR in 13 of 15 categories. By means of this method, it is not only possible to generate real-looking STIR images but, above all, to generate images that depict reality.

For limitations, the datasets contained only MRI examination from a single vendor, therefore, the network may not be generalizable to other MRI vendors [25,26]. Furthermore, our method was validated for only 15 different categories of pathologies / findings; it is still uncertain whether the vSTIR is equal to rSTIR in demonstrating other pathologies. A true 3D or 2.5D network may be able to employ more local information into the generation of the vSTIRs, thereby increasing the output quality.

## 5. Conclusions

In conclusion, our study underlines the potential of a cGAN for generating STIR images from T1 and T2 images. Overall, we had very good results in the similarity of the vSTIR to rSTIR images and in displaying the most important pathologies. This may lead to reduced MR scanning time and to a reduced re-scan rate. In the next step, our database must be increased and validated on a multi-center basis to avoid overfitting to a single vendor.

## Figures and Tables

**Table 1:** Distribution of the examinations to the different MR scanners.

Scanner	Aera	Avanto	Sonata	Symphony	Skyra
*n*	521	188	7	16	21
Field strength	1.5T	1.5T	1.5T	1.5T	3T

**Table 2:** Baseline characteristics of the Train and Test set.

Set	Studies	Patients	Sex (Male/Female)	Mean Age
Train	612	514	284/328	60.3
Test	141	133	78/63	60.1

**Table 3:** Distribution of pathologies across the validation cohort. Each scan in which a pathology occurs once or several times was counted as 1.

Pathology	Bone Metastases	Myelopathy	Acute Vertebral Fracture	Spondylodiscitis	Epidural Abscess	Intraspinal Mass	Muscular Lesion
*n*	41	24	39	42	24	33	26

**Table 4:** Mean error and the statistical significance for each rater.

Rater	Mean Error (in %)	*p*-Value (Fisher Test)
1	41 %	0.008
2	44 %	0.06
3	58 %	0.992
4	48 %	0.336
5	39 %	0.001
6	45 %	0.101

**Table 5:** Results of all equivalence tests.

	Mean STIR	Mean vSTIR	Interrater Agreement STIR	Interrater Agreement vSTIR	Significance (*p*-Value) for Difference between Interrater Agreements	Significance (*p*-Value) for Equivalence (*δ* = 0.10)	Statistical Equivalent (*δ* = 0.10)?
Hyperintense Vertebral Body	32% (none) 39% (low) 29% (high)	37% (none) 39% (low) 24% (high)	0.61 (*p* < 0.01)	0.59 (*p* < 0.01)	0.37	0.08	No
Vertebral Bone Fractures	69% (none) 27% (low) 4% (high)	66% (none) 30% (low) 4% (high)	0.54 (*p* < 0.01)	0.60 (*p* < 0.01)	0.32	0.01	Yes
Hyperintense Discs	67% (none) 24% (low) 9% (high)	71% (none) 20% (low) 9% (high)	0.39 (*p* < 0.01)	0.35 (*p* < 0.01)	0.23	0.01	Yes
Real STIR	57%	51%	0.02 (*p* = 0.70)	<0.01 (*p* = 0.96)	0.66	0.42	No
Intraspinal Mass	17%	12%	0.34 (*p* < 0.01)	0.24 (*p* < 0.01)	0.03	0.038	Yes
Myelopathy	19%	17%	0.63 (*p* < 0.01)	0.66 (*p* < 0.01)	0.57	0.001	Yes
Muscular Edema	26%	26%	0.52 (*p* < 0.01)	0.43 (*p* < 0.01)	0.07	0.001	Yes
Spondylodiscitis	21%	22%	0.66 (*p* < 0.01)	0.59 (*p* < 0.01)	0.13	0.003	Yes
Epidural Abscess	12%	9%	0.54 (*p* < 0.01)	0.41 (*p* < 0.01)	<0.01	0.003	Yes
Muscular Abscess	8%	7%	0.31 (*p* < 0.01)	0.39 (*p* < 0.01)	0.13	0.004	Yes
Bone Metastases	22%	17%	0.64 (*p* < 0.01)	0.56 (*p* < 0.01)	0.07	0.055	No
Intraspinal Neoplasia	9%	6%	0.45 (*p* < 0.01)	0.45 (*p* < 0.01)	0.93	<0.001	Yes
Acute Traumatic Fracture	13%	13%	0.79 (*p* < 0.01)	0.58 (*p* < 0.01)	<0.01	<0.001	Yes
Pathological Fracture	8%	7%	0.46 (*p* < 0.01)	0.19 (*p* < 0.01)	<0.01	<0.001	Yes
Benign Bone Neoplasia	2%	2%	0.12 (*p* < 0.01)	0.43 (*p* < 0.01)	<0.01	<0.001	Yes
Normal	16%	17%	0.70 (*p* < 0.01)	0.60 (*p* < 0.01)	0.03	0.004	Yes

## Data Availability

The data presented in this study are available on request from the corresponding author. The data are not publicly available for data protection reasons.

## Supplementary Materials

The following are available online at https://www.mdpi.com/article/10.3390/diagnostics11091542/s1, Table S1. TE and TR relaxation times in ms (mean ± standard deviation), Table S2. Slice thickness in mm (mean ± standard deviation), Table S3. Pixel spacing in mm (mean ± standard deviation).

## Abbreviations

cGAN	conditional Generative Adversarial Network
GAN	Generative Adversarial Network
MRI	Magnetic Resonance Imaging
rSTIR	real Short Tau Inversion Recovery
STIR	Short Tau Inversion Recovery
vSTIR	virtual Short Tau Inversion Recovery
