# Supplementary material for: Generating Virtual Short Tau Inversion Recovery (STIR) Images from T1- and T2-Weighted Images Using a Conditional Generative Adversarial Network in Spine Imaging
Source: Diagnostics (Basel). 2021 Aug 25;11(9):1542. doi: 10.3390/diagnostics11091542 (PMC8467788; doi:10.3390/diagnostics11091542)
Supplement: Supplementary file 1 [file diagnostics-11-01542-s001.zip › diagnostics-1296971-supplementary.pdf]

Supplementary Material

Supplementary Table S1. TE and TR relaxation times in ms (mean ± standard deviation).

Scanner	T1 TE	T2 TE	STIR TE	T1 TR	T2 TR	STIR TR
**Aera**	11.5 ± 0.9	107.0 ± 2.3	64.3 ± 5.6	533.9 ± 81.8	5207.0 ± 1011.6	4378.1 ± 672.4
**Avanto**	13.1 ± 1.1	106.3 ± 4.9	72.6 ± 6.1	590.2 ± 64.8	4293.2 ± 568.3	5880.1 ± 973.2
**Skyra**	11.2 ± 1.8	103.8 ± 9.8	64.0 ± 7.4	1032.0 ± 959.7	4546.1 ± 1250.1	4030.1 ± 1040.2
**Sonata**	12.0 ± 0.0	108.0 ± 2.6	75.4 ± 1.5	488.9 ± 52.0	5397.1 ± 392.2	5674.3 ± 790.6
**Symphony**	13.8 ± 0.8	111.2 ± 0.7	107.5 ± 4.3	539.0 ± 56.4	4831.2 ± 1137.2	4481.2 ± 777.9

Supplementary Table S2. Slice thickness in mm (mean ± standard deviation).

Scanner	T1 Thickness	T2 Thickness	STIR Thickness
**Aera**	2.7 ± 0.5	2.7 ± 0.5	2.7 ± 0.5
**Avanto**	3.1 ± 0.1	3.1 ± 0.1	3.1 ± 0.1
**Skyra**	2.8 ± 0.5	2.8 ± 0.5	2.8 ± 0.5
**Sonata**	2.9 ± 0.4	2.9 ± 0.4	2.9 ± 0.4
**Symphony**	2.8 ± 0.4	2.7 ± 0.5	2.8 ± 0.4

Supplementary Table S3. Pixel spacing in mm (mean ± standard deviation).

Scanner	T1 Spacing	T2 Spacing	STIR Spacing
**Aera**	0.66 ± 0.06	0.66 ± 0.06	0.66 ± 0.07
**Avanto**	0.72 ± 0.05	0.72 ± 0.05	0.72 ± 0.05
**Skyra**	0.66 ± 0.06	0.64 ± 0.07	0.66 ± 0.08
**Sonata**	0.73 ± 0.09	0.72 ± 0.08	0.72 ± 0.08
**Symphony**	0.74 ± 0.1	0.74 ± 0.1	0.74 ± 0.1
